# Correction: Gurunathan, S. et al. Review of the Isolation, Characterization, Biological Function, and Multifarious Therapeutic Approaches of Exosomes. *Cells* 2019, *8*, 307

**DOI:** 10.3390/cells10020462

**Published:** 2021-02-22

**Authors:** Sangiliyandi Gurunathan, Min-Hee Kang, Muniyandi Jeyaraj, Muhammad Qasim, Jin-Hoi Kim

**Affiliations:** Department of Stem Cell and Regenerative Biotechnology, Konkuk University, 1 Hwayang-Dong, Gwangin-gu, Seoul 05029, Korea; pocachippo@gmail.com (M.-H.K.); muniyandij@yahoo.com (M.J.); qasimattock@gmail.com (M.Q.)

In the original review [1], we found that there is a mistake in Figure 3 as published. Figure 3 has incorrect labelling and reversed orientation. Figure 3 relates to exosomes derived from silver nanoparticle-treated SH-SY5Y cells, as described in the legend. The corrected Figure 3 appears below. The authors apologize for this error and state that this does not change the content or scientific conclusions of the review in any way. 

The updated figure is shown below.

Correct Figure 3:

**Figure 3:**
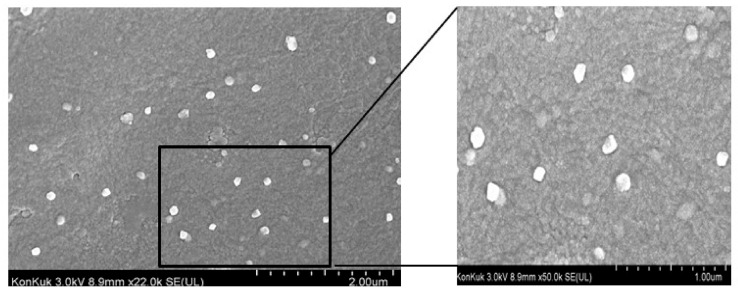
Silver nanoparticles induce biogenesis and secretion of exosomes in SH-SY5Y cells cultured with serum-free medium.
